# Barriers and enablers of kangaroo mother care implementation from a health systems perspective: a systematic review

**DOI:** 10.1093/heapol/czx098

**Published:** 2017-08-24

**Authors:** Grace Chan, Ilana Bergelson, Emily R Smith, Tobi Skotnes, Stephen Wall

**Affiliations:** 1 Medicine Critical Care, Boston Children’s Hospital, Boston, MA, USA,; 2 Department of Global Health and Population, Harvard T.H. Chan School of Public Health, Boston, MA, USA and; 3 Saving Newborn Lives, Save the Children, Washington, DC, USA

**Keywords:** Health systems, health facilities, health professionals

## Abstract

Kangaroo Mother Care (KMC) is an evidence-based intervention that reduces neonatal morbidity and mortality. However, adoption among health systems has varied. Understanding the interaction between health system functions—leadership, financing, healthcare workers (HCWs), technologies, information and research, and service delivery—and KMC is essential to understanding KMC adoption. We present a systematic review of the barriers and enablers of KMC implementation from the perspective of health systems, with a focus on HCWs and health facilities. Using the search terms ‘kangaroo mother care’, ‘skin to skin (STS) care’ and ‘kangaroo care’, we searched Embase, Scopus, Web of Science, Pubmed, and World Health Organization Regional Databases. Reports and hand searched references from publications were also included. Screening and data abstraction were conducted by two independent reviewers using standardized forms. A conceptual model to assess KMC adoption themes was developed using NVivo software. Our search strategy yielded 2875 studies. We included 86 studies with qualitative data on KMC implementation from the perspective of HCWs and/or facilities. Six themes emerged on barriers and enablers to KMC adoption: buy-in and bonding; social support; time; medical concerns; training; and cultural norms. Analysis of interactions between HCWs and facilities yielded further barriers and enablers in the areas of training, communication, and support. HCWs and health facilities serve as two important adopters of Kangaroo Mother Care within a health system. The complex components of KMC lead to multifaceted barriers and enablers to integration, which inform facility, regional, and country-level recommendations for increasing adoption. Further research of methods to promote context-specific adoption of KMC at the health systems level is needed.

Key Messages

- Kangaroo mother care (KMC) is an effective, evidence-based intervention to reduce neonatal morbidity and mortality, and the health system plays an essential role in the scale up and adoption of KMC.
- Healthcare workers (HCWs) and health facilities face unique barriers to implementing KMC, especially in areas of social support, leadership buy-in, and access to training.
- Clear and consistent communication between HCWs and health facility leadership is an important enabler of successful KMC adoption.
- Analysis of KMC adoption through a health system perspective, with a focus on HCWs and health facilities, should inform strategies for health system adoption of KMC at the facility, regional, and national levels.

## Introduction

The scale up of evidence-based newborn interventions such as kangaroo mother care (KMC) is often influenced by the strength of health systems. KMC includes early and continuous skin-to-skin contact (SSC) between the newborn and caregiver, exclusive breastfeeding, early discharge from health facilities, and supportive care and follow up (World Health Organization 2003). Among preterm and low-birth weight (<2000 g) newborns, the clinical efficacy and health benefits of KMC has been widely demonstrated in multiple settings (Lawn *et al.* 2010; Bergh *et al.* 2014). However, implementation of KMC has been inconsistent across different health systems with several factors affecting its adoption, including availability of health workers and resources, absence of health worker training, and lack of government support (Bergh *et al.* 2014; Vesel *et al.*, 2015)

To maximize the effectiveness of a health system, all components of the system—leadership and governance, financing, health workforce, technologies, information and research, and service delivery—must function in an integrated manner that recognizes the inter-dependence of each part of the system (World Health Organization 2007). Implementation of an intervention such as KMC relies on a well-functioning health system. For example, healthcare workers (HCWs) are needed to disseminate KMC knowledge and train caregivers to practice KMC. In a global review of KMC barriers and facilitators, results showed that health facilities, where KMC is often initiated, require a minimum level of leadership, financing, and information in order to successfully deliver KMC (Chan *et al.* 2016). In this systematic review, we further explore the barriers and enablers of KMC implementation specifically from the perspective of health systems, with a focus on HCWs and health facilities.

## Materials and methods

**Figure 1.**
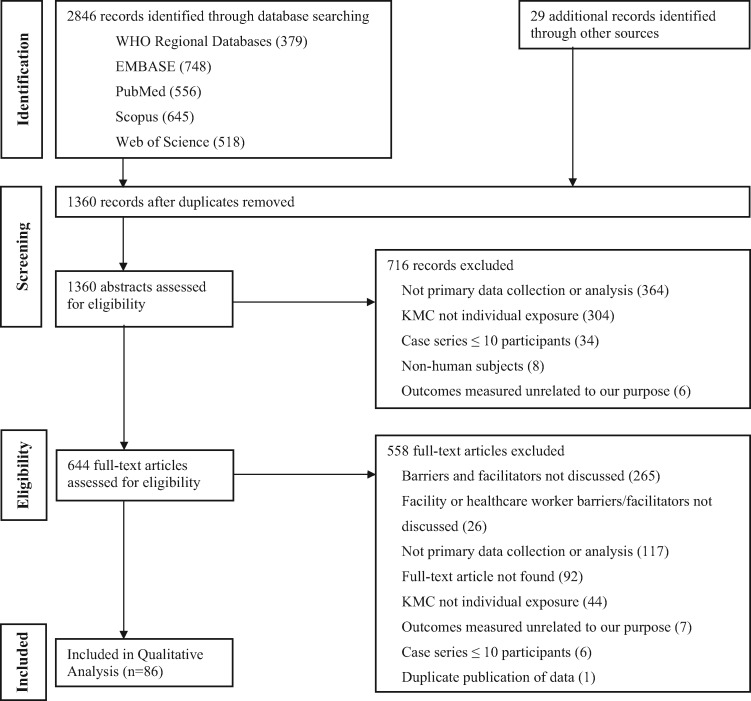
Flowchart for study selection

Using the search terms ‘KMC’ or ‘kangaroo care’ or ‘STS care’, we searched Pubmed, Embase, Web of Science, Scopus and World Health Organization Regional Databases from January 1, 1960 to August 19, 2015. In addition, we hand searched reference lists of included articles, published systematic reviews, and requested data from programmes implementing KMC such as Save the Children. Studies were included if they contained primary data on barriers or enablers of KMC implementation. Studies were excluded if they did not specifically discuss barriers or enablers from a HCW or health facility perspective (Figure 1).

Screening and data abstraction were conducted by two independent reviewers using standardized forms to identify factors of KMC implementation. Study quality was evaluated based on selection bias, data collection and analysis methodology, generalizability, and ethics (Kuper *et al.*, 2008). Using the qualitative analytical software NVivo (QSR International, Melbourne, Australia), two researchers indexed and annotated the data. Narratives were constructed and categorized into matrices by theme.

## Results

Following a screen of 2875 articles, 86 articles were eligible for inclusion. Of the 86 included studies, 53 studies (61.6%) were published in 2010–15, 53 (61.6%) had a sample size of <50, nearly half of the studies were based on interview or survey data (47.7%) and approximately one-third of the studies occurred in the Americas (32.6%). Most studies were based in a Neonatal Intensive Care Unit (NICU) (32.6%), and 9.3% of studies were community or population-based (outside of health facilities or hospitals and usually involving community health workers [CHWs]). The characteristics of the included studies are provided in Table 1, with full details in Supplementary Table S1.

**Table 1.** Characteristics of included studies (*n* = 86)

	*n*	%
**Year**		
2010–15	53	61.6
2000–09	30	34.9
1988–99	3	3.5
**Sample Size**		
< 50	53	61.6
50 to < 100	10	11.6
100 to < 200	10	11.6
≥200	13	15.1
**Study type**		
Survey or interview	41	47.7
Facilities evaluation	15	17.4
Randomized control trial	7	8.1
Cohort study	2	2.3
Other	20	23.3
Pre-post	1	1.2
**WHO region**		
Americas	28	32.6
Africa	20	23.3
Europe	18	20.9
Southeast Asia	10	11.6
Eastern Mediterranean	3	3.5
Western Pacific	3	3.5
Multiple regions	3	3.5
Missing	1	1.2
**NMR (deaths per 1000 live birth)**		
<5	32	37.2
5 to < 15	17	19.8
15 to < 30	28	32.6
≥30	3	3.5
Missing	6	7.0
**Setting (rural or urban)**		
Urban	32	37.2
Urban and rural	13	15.1
Rural	4	4.7
Missing	37	43.0
**Population source**		
Health facility	50	58.1
NICU or stepdown unit	28	32.6
Community or population-based surveillance	8	9.3
**Gestational Age**		
Preterm 34 to < 37 weeks	9	10.5
All gestational ages	10	11.6
Very preterm <34 weeks	8	9.3
Mixed preterm and very preterm <37 weeks	3	3.5
Full term ≥ 37 weeks	3	3.5
Missing	53	61.6
**Birth weight**		
LBW 1500 to < 2500 g	9	10.5
All birth weights	11	12.8
Mixed low and very LBW <2500 g	2	2.3
Very LBW <1500 g	2	2.3
Missing	62	72.1

We visualize a health system as a complex system with key components (e.g. HCWs and health facilities) with unique interests and values that contribute to the uptake of health interventions (Atun *et al.* 2010). HCWs ensure that KMC is implemented within the facility. In some facilities nurses are responsible for conducting KMC with preterm infants. But for the most part, HCWs are responsible for educating both the parents and the facility leadership to convince both of the importance of KMC, and to ensure that KMC is provided in their department for eligible newborns. Facilities include the structural building and the leadership team that runs the facility. The location of the facility and the available resources play an important role in whether KMC takes place inside or outside of the facility (e.g. follow up KMC after discharge by members of the health facility). Furthermore, the acceptance of KMC by facility leadership plays a role in resource allocation and implementation of KMC within the facility.

### Barriers and enablers to KMC

We identified six themes that describe the barriers and enablers encountered by HCWs and facilities (Table 2): buy-in (acceptance of KMC and its benefits), social support and empowerment (encouragement and aid in performing KMC), time (time to train and provide KMC), medical concerns (health status of mother or infant), access (availability of training and resources), and cultural norms (sociocultural context of newborn care and facility policies). The themes, organized by barriers and enablers (at the HCW and facility level) to KMC adoption, are presented in Table 2 and are discussed below.

**Table 2.** Matrix of barriers and enablers for HCWs and health facilities

		Buy-in	Support and Empowerment	Time	Medical concerns	Access	Cultural norms
**HCWs**	**Enablers**	Experience with KMC Nurses were more likely to perform KMC if they believed it worked	**Management:** Management mobilization of resources Nurse involvement in care related decision making	**Workload** Some nurses reported that KMC did not increase the amount of time they spent on each patient	Practicing securing catheters lowered nurses’ concerns Nurses with 5 or more years of experience more likely to implement KMC	Expanding training to other healthcare personnel besides nurses	Some HCWs advised mothers to delay bathing so infant would not get cold
			**Other HCWs:** Multiple health worker support facilitated SSC—nutrition workers, CHWs and clinical workers				
	**Barriers**	Nurses believe KMC based on perception and not scientific fact Inconsistent application of KMC within facilities and among HCWs Concerns on the stability of the infant	**Management:** Lack of leadership and support from management Felt newborn care was not a priority in the health system	**Workload** Training mothers to do SSC would take additional time out of health workers’ schedules, increase their workload, and reduce time with other critical patients	Did not believe KMC was safe for LBW newborns Staff not trained in preterm care	KMC training not part of a broader healthcare training curriculum Poor training lead to conflicting knowledge on time and duration of SSC	**Traditional Newborn Care** Bathing practices and wrapping infants soon after birth delayed SSC In warm climates staff did not believe hat and socks were necessary
			**Other caregivers:** Some HCWs considered parents and visitors as a barrier Limited communication between HCWs				
**Health Facilities**	**Enablers**	Companions for mothers promoted KMC Posters of KMC in the facility	Use of technology Use of KMC guidelines	Greater or unlimited visitation time enhanced support from family and promoted KMC KMC ward	Shorter crying times in response to pain with KMC compared with incubator care	Access to private space/privacy screens Relaxed atmosphere with dim lighting	Include KMC in health facility statistics
	**Barriers**	Management reluctance to allocate space for SSC High leadership turnover	KMC protocols perceived as inflexible	Shortage of staff nurses limited parental access and shortened visitation time. The shorter the visitation period was, the more of an interference staff thought parents were Visitation policies were difficult due to strained communication between parents and staff. Visitors were an obstacle to breastfeeding and KMC performance	Few NICUs had written KMC protocols No checklist for KMC admission procedures. Follow-up and discharge procedures not well structured	**Space:** Lack of privacy Space limitations induced discharge within hours Crowding and insufficient space in the NICU. **Allocation:** Staff need to bargain with managers to increase and maintain resources for newborn care KMC was not budgeted for, and resources were mismanaged	No record of SSC Difficulty adapting/teaching electronic medical records for KMC Implementing continuous KMC was difficult. Many facilities reported performing continuous KMC, but few actually practiced it

KMC, kangaroo mother care; HCW, healthcare worker; SSC, skin-to-skin contact; LBW, low birth weight.

#### Buy-in

##### Healthcare workers

Several factors affected HCW buy-in of KMC. Many healthcare staff believed newborn care was not a high priority at their facility.(Bergh *et al.* 2007) Furthermore, KMC was perceived as a disadvantage by nurses because the mother needed to remain in the hospital (Solomons and Rosant 2012). In some cases, KMC was cited as the ‘poor man’s alternative’ for developing countries and considered to be a sub-standard method of care (Charpak and Ruiz-Pelaez 2006). For skeptical nurses who did not believe in KMC (often from lack of experience), support from more experienced nurses allowed them to see the benefits of KMC and facilitated nurse buy-in (Eichel 2001; Johnson, 2007).

*I find it a great joy when the mums do hold the baby against their chest … irrespective of whether it’s a primigravida or a multigravida. You get the same buzz out of it and so do the dads.* (Nurse) (Chia *et al.* 2006)

##### Facilities

Staff shortages, high turnover of staff, and an insufficient number of trained HCWs inhibited buy-in of KMC at health facilities. When a particular HCW has served as the primary promoter of SSC in a facility, if they departed the hospital, it increased the difficulty of educating other staff members on the practice of SSC (Lee *et al.* 2012). At some institutions, researchers heard reports of practicing ‘intermittent KMC in our hospital’, but observed no individuals practicing KMC during surprise visits with explanations of ‘staff shortages’ or lack of presence of KMC supporters (Bergh and Pattinson 2003). Even with a presence of KMC supporters, an insufficient number of staff inhibited KMC:

*We’re still kind of stumbling a little bit because of our lack of manpower to move forward with a lot of our things. I think the intent and the will is there, just we require more team members.* (Health facility staff) (Lee *et al.*, 2012)

However, in some Ugandan facilities, KMC acceptance was promoted through posters with directions or with pictures of previously admitted mothers performing KMC (Bergh *et al.* 2012b). Several facilities in Uganda allowed mothers to have companions, which aided KMC promotion at the facility level and strengthened moral support for mothers (Bergh *et al.* 2012b).

#### Social support and empowerment

##### Healthcare workers

HCWs were unable to implement KMC without support from parents and facility leadership. Lack of parental participation in KMC was observed in one facility. For example, parents did not assist with transferring the infant in and out of the incubator in order to perform KMC; therefore nurses had to perform these KMC activities, impeding their ability to continue their other responsibilities (Strand *et al.* 2014). In addition, nurses sought support from management through ‘educational programmes’ ‘adequate staffing’ and ‘encouragement’ (Johnson 2007). To further empower nurses to perform KMC, one NICU in the United States employed ‘Pioneer Nurses’, who were nurses with experience in KMC that would teach less experienced nurses about the benefits of SSC by helping them facilitate SSC between mothers and infants (Eichel 2001).

##### Facilities

There was resistance to change by staff who were wary of implementing new KMC protocols when they were concerned about the welfare of babies (Eichel 2001). Although some staff believed KMC guidelines would be helpful, others believed they would cause inflexibility (Chia *et al.* 2006). Studies showed the use of technology increased support for KMC within facilities. For example in Ghana, HCWs attended KMC workshops and then received cell phone messages encouraging KMC implementation. Feedback from HCWs was positive:

*Glad for your constant reminders. We are on it* and *KMC is working. Expecting to hear more from u*. (Healthcare workers) (Bergh *et al.*, 2012d)

#### Time

##### Healthcare workers

There was a common belief among nurses that training the mothers to do SSC would take more time than they had available, and nurses were concerned about not having time to attend to their other newborn patients in the NICU (Charpak and Ruiz-Pelaez 2006; Engler *et al.* 2002). In certain facilities, when there was an overabundance of patients, KMC mothers were the lowest priority for HCWs (Bergh *et al.* 2012a). In some cases, staff did not have time to learn new KMC protocols (Engler *et al.* 2002).

“*KC takes too much work, too much time”* and *“[I am] not willing to take extra time with the family that KC requires*.” (Healthcare Worker) (Engler *et al.* 2002)

In other cases however, HCWs did not believe that KMC increased their workload, or decreased their time spent on other infants.

*KMC was effective in taking care of the vitals and temperature regulation of the LBWI [low birth weight infant] and it was worth putting efforts to promote and continue KMC in the unit*. (Healthcare worker) (Parmar *et al.* 2009)

##### Facilities

Shortage of staff nurses, limited parental access, and shortened visitation time presented an obstacle to KMC uptake. Specifically, communication between staff and parents was strained in facilities with limited visitation hours. Parents were unhappy with shorter visitation times, as were staff, who found parents to be a greater interference when visitation was limited (De Vonderweid *et al.* 2003). Some staff did not feel comfortable taking time to assist their colleagues in transferring infants to KMC, and if visitation times were longer, KMC was inhibited by mothers foregoing breastfeeding because others were in the room (Ferrarello 2014).

*… visitors in the room impact breastfeeding, as well. Sometimes hours and hours go by and the mother won’t feed their babies or pump or anything because people are there.* (Nurse) (Ferrarello 2014)

Thus, some studies showed that visitation time negatively impacted KMC implementation from both a health provider and parent standpoint.

Conversely, some nurses and healthcare staff thought that long visitation hours facilitated KMC implementation because it would allow for fathers and other family to come in and support the mothers (Eichel 2001; De Vonderweid *et al.* 2003). Additional enablers of care outside of visitation time included a KMC ward to promote mothers being with their infants fulltime (Hennig *et al.* 2006), or having a flexible stay at a facility where mothers could go home and then come back to the facility to practice KMC (Toma *et al.* 2007).

#### Medical concerns

##### Healthcare workers

Many HCWs had a fear of hurting the baby during SSC while the baby was still attached to wires and cords (Engler *et al.* 2002). Additionally, nurses were hesitant to use KMC for infants with catheters, whether intravenous, arterial or umbilical, or for intubated infants (Engler *et al.*, 2002; Flynn and Leahy-Warren 2010; Lee *et al.* 2012).

“…*being afraid something will go wrong and I will be blamed for it”* and *“…not [being] sure KC is safe”* (Nursing Staff Members) (Engler *et al.* 2002)

Nurses were also not comfortable recommending KMC for infants under 1000 g (Stikes and Barbier 2013). There was a lack of sufficient training for healthcare providers and no specific programmes and facilities for care of preterm babies despite newborn care being lauded as a national health policy priority in some countries, such as Uganda (Waiswa *et al.* 2010). Practicing strategies for securing umbilical catheters may alleviate the concerns of nurses about KMC for babies with them (Engler *et al.* 2002).

##### Facilities

Food was often unavailable at facilities for mothers, therefore mothers were unable to breastfeed their newborns, left the hospital early, or depended on relatives for food (Bergh *et al.* 2013). In addition, NICUs within facilities disagreed over the definition of clinical stability (Lee *et al.* 2012). However, in some facilities, KMC provided health benefits for the infants. One study found higher breast feeding rate at discharge in those facilities where continuous SSC and breastfeeding were encouraged (Boo and Jamli 2007). One study found that infants with KMC had shorter crying times after a heel stick, a painful stimulus, than did those with incubator care (Kostandy *et al.* 2008).

#### Access to training and resources

##### Healthcare workers

Access to adequate and consistent training presented a barrier for HCWs to implement KMC. Inadequate training in KMC led nurses even within the same facility to have conflicting knowledge about the time and duration of SSC contact (Chia *et al.* 2006; Lemmen *et al.* 2013). In a facility in Australia the percentage of nurses trained in KMC was only ∼50% (Chia *et al.* 2006). To facilitate access to KMC training, online work-share technologies at one facility in the United States allowed communication between staff who were unable to find a common time for training (Haxton *et al.* 2012). In South Africa, hospitals that received visits from facilitators discussing KMC via workbooks, videos, and teaching posters were more successful at implementing KMC than facilities that did not have KMC facilitator visits (Pattinson *et al.* 2005).

*I think they [nurses] need to have knowledge of it…myself included, I probably need more knowledge…Certainly it needs to be revisited a lot of the time so that the staff can see the importance of it.* (Nurse) (Chia *et al.* 2006)

This quote from Chia *et al*. demonstrates that additional knowledge provided through KMC training would have served to emphasize the importance of KMC implementation.

##### Facilities

In some cases, lack of ambulatory services needed to transport mothers was considered to be barriers to accessing as well as conducting follow-up at the facility (Toma 2003; Blencowe *et al.* 2009). Long distances, no public transportation options and poor road conditions inhibited facility access as well (Bergh *et al.* 2012c). Structurally, crowding and insufficient space in facilities presented a barrier to KMC, hastening discharge (one study reported a discharge in under two hours) or restricting visitation policies because of staffing shortages and space concerns (Bergh *et al.* 2012d). If mothers are unable to visit their babies due to restricted visitation policies, KMC cannot be performed. Mothers are also less likely to stay in the hospital to practice KMC if their families cannot visit them. In addition, insufficient privacy due to space concerns and lack of privacy screens coupled with discomfort with being undressed in the presence of strangers acted as a KMC barrier (Blomqvist *et al.* 2013; Nahidi *et al.* 2014) One mother expressed:

*There were always people around. It is harder (to be skin to skin) when there are other people coming in. Private rooms will help.* (Mother) (Ferrarello, 2014)

Private rooms or areas that allowed both parents to stay also contributed to successful KMC implementation, (Neu 1999; Stikes and Barbier 2013) and some facilities did include privacy screens in the labor ward (Namazzi *et al.* 2015). In addition, mothers preferred rooms with dimmer lighting, as fluorescent lights were bothersome (Johnson 2007; Nyqvist and Kylberg 2008). In similar cases, managerial staff reallocated resources donated for KMC to other departments and higher paying users (Bergh *et al.* 2012c), and staff experienced difficulty in bargaining with managers to allot more resources to newborn care services (Furlan *et al.* 2003), with KMC. National maternal and child health policies did not include budgets for KMC, and guidelines for district or local budgeting for KMC services were needed (Save the Children—Saving Newborn Lives Program 2011; Bergh *et al.* 2012a). 

#### Cultural norms

##### Healthcare workers

Cultural norms displayed by HCWs regarding newborn clothing and discharge from the hospital were barriers to KMC adoption. In one hospital, staff did not believe that the infants needed to wear caps or socks in hot climates, and therefore did not incorporate these items into the KMC package needed to provide follow-up of the KMC infant (Charpak and Ruiz-Pelaez 2006). Regarding discharge, one study noted infants were traditionally discharged on average within 6 days of birth, making it more difficult, operationally and culturally, to keep mother/infant pairs in the hospital for extended durations of time (Blencowe *et al.* 2009). Additionally, in some cultures, newborns were immediately bathed after delivery preventing immediate SSC with the mother (Hill *et al.* 2010).

*Even in the hospitals babies are bathed immediately after delivery so why do I [referring to the interviewer] want them to delay the bathing…Babies are normally bathed shortly after birth because it will help them feel clean and healthy*.(Mother) (Hill *et al.* 2010)

However, in a study in India, 85.2% of Accredited Social Health Activists advocated delayed bathing and 64% of mothers waited until at least 48 hours after delivery to bathe their infant (Sinha *et al.* 2014), thus creating the possibility of immediate SSC.

##### Facilities

Documentation of KMC practice varied between facilities. In some facilities, KMC was performed but never recorded or lacked detail, which prevented the facility from accurately monitoring KMC practices (Lee *et al.* 2012). However, some studies reported that the use of site assessment tools and performance standards facilitated implementation. One facility modified their electronic medical records to enable nurses to document the initiation and duration of KMC (Haxton *et al.* 2012).

### Interactions between HCWs and facilities

We analysed the different perspectives from each level of the health system to create more meaningful and impactful recommendations of how to streamline KMC (Table 3).

**Table 3:** Interactions among HCWs and health facilities

Key actors	Other actors	Themes
**HCWs**	**HCWs**	**Communication**: The current approach to SSC is siloed with little communication between staff. Furthermore, training and knowledge on the method is usually exclusive to NICU and maternity units, so there is not greater buy in of other HCWs in the practice.
	**Facility**	**Structural Resources**: Lack of resources (beds, wraps, etc.) make implementation of SSC hard for HCWs. Sometimes they have to find creative ways to implement KMC despite lack of resources.
		**Staffing**: Staffing shortages within facilities put stress on HCWs who take on greater caseloads. Also frequent staff rotations give HCWs very little time to practice and use KMC after they learn it. Lack of practice can lead to the degradation of skills and the frequent rotations mean that the facility has to continuously teach KMC to staff which results in a lack of experts within the facility that champion KMC.
**Facilities**	**HCWs**	**Leadership**: Nurses and other staff performing KMC often feel like they have to negotiate with leadership and management to get more space and resources for KMC. Leadership doesn’t always allocate the appropriate resources or believe in the practice of KMC.
		**Training**: Hard to coordinate HCWs schedules to find a time to train them in SSC/KMC facility policies. One solution found to this problem was the use of online virtual trainings that could be done individually.
		**Buy-In**: In some facilities leadership and management do not believe in KMC since they are not included in KMC training. Therefore, KMC is not seen as a hospital priority and limited resources are redistributed to other departments/patients.
	**Facilities**	**Communication**: There is very little communication between facilities. Coordination on guidelines and modifications for facilities that see similar populations can improve continuity of care for mothers transferring hospitals and can allow for more countrywide and culturally appropriate modifications to KMC policy.

KMC, kangaroo mother care; SSC, skin-to-skin contact.

#### HCWs with each other

Lack of communication between HCWs created a siloed approach to KMC implementation (Lee *et al.* 2012). Although NICU nurses were educated in KMC it was rare for other HCWs throughout the hospital to know much about KMC (Save the Children—Saving Newborn Lives Program 2011). There was very little communication with or training of HCWs that worked outside of the hospital, such as CHWs and midwives. The importance of communication between health workers to implement KMC was demonstrated in Malawi where KMC was taught to non-facility staff (Bergh *et al.* 2012a). Another study describes communication with CHWs as an enabler as well:

*We found that CHWs were not knowledgeable on STS [skin to skin] care or KMC, but once we described the procedures, they showed willingness to promote them if trained.* (Researcher) (Waiswa *et al.* 2010)

#### HCWs and facilities

In terms of the facility, staffing shortages, lack of leadership buy-in, and lack of space all played a role in the ability for HCWs to implement KMC (Johnson 2007; Bergh *et al.* 2012a). In many cases, HCWs had to negotiate with facility leadership to get the resources necessary to allow parents to participate in KMC (i.e. chairs, beds, and private spaces) (Bergh *et al.* 2012b) The HCWs were limited without the proper resources, yet they were often trained in KMC and asked to implement it within the facility. HCWs trained in KMC functioned like pioneers in the hospital who had to convince not only the mothers of the benefits of KMC, but often the facility leadership as well, who were not required to attend trainings. Frequently, the HCWs had to find a time to train both mothers and leadership amidst staffing shortages (Save the Children—Saving Newborn Lives Program 2011). In some instances, nurses incorporated KMC into huddles, thus promoting KMC discussions to come from coworkers and not senior management (Lee *et al.* 2012).

#### Facilities and other facilities

Even though guidelines were uniform at a national level, some facilities made adjustments to accommodate barriers within their specific facility (Save the Children—Saving Newborn Lives Program 2011). However, different policies between hospitals posed as a barrier to KMC implementation and continuity of care for mothers changing facilities. Without communication and coordination among the facilities, it was hard to ensure that the facilities were implementing the guidelines consistently (Bergh *et al.* 2012c). Additionally, there was limited interaction regarding effective and ineffective approaches and possible work around alternatives to cultural and other barriers (Bergh *et al.* 2012d; Lee *et al.* 2012).

## Discussion and recommendations

To facilitate KMC implementation, we present facility-, regional- and country-level recommendations based on evidence from the literature (additional details are available in Supplementary Table S2).

### Facility-level recommendations

Within facilities, it is necessary to strengthen KMC protocols (Bergh *et al.* 2012a,c). KMC should be practiced systematically, and checklists for mothers and infants should be initiated to ensure continuity of care. Furthermore, ‘succession planning’ should be used in order to have an adequate number of trained staff available on a regular basis (Bergh et al. 2012e), and facility staff should clearly understand policies on criteria for discharging KMC infants (Bergh *et al.* 2012b). Additionally, to implement a revised KMC policy within facilities, it is important to support and trouble shoot problems as they appear. Continued education of staff and parents is necessary (Hendricks-Munoz *et al.* 2010) and providing a better liaison with antenatal care services to endorse KMC could contribute to better preparation of caregivers and training of HCWs (Bergh *et al.* 2012a).

Last, facilities often interpreted guidelines as malleable and changed them according to their specific situation (Save the Children—Saving Newborn Lives Program 2011). However, different policies between hospitals posed as a barrier to KMC implementation and continuity of care for mothers changing facilities. Therefore, facilities and policy makers need to come together to discuss more realistic KMC guidelines and potential modifications for particular situations within countries (Bergh *et al.* 2012d).

### Regional-level recommendations

In some regions, the government provided KMC trainings for HCWs (Sacks *et al.* 2013). However, while HCWs from most facilities would have liked to participate in the trainings, the distance of the trainings and staff shortages within the hospital facilities limited the number of staff that could attend. Although the policy makers were trying to help, they did not anticipate these barriers to healthcare staff attendance (Bergh *et al.* 2007).

In another tactic to increase uptake of KMC, some policy makers focused on a uniform expansion of the policy. However, this may not have been the correct approach. For facilities that rarely had preterm infants or any infants that meet the KMC criteria, it was an inefficient use of resources and money to train their staff in KMC procedures. Therefore, targeted expansion of KMC to facilities that often deliver preterm babies and expansion to CHWs that help deliver babies in the home may be a better use of resources (Bergh *et al.* 2012c).

A study in Mali serves as a successful example of reinvigoration of KMC. Two tiers of involvement were proposed in facilities and in the community. Facilities included refresher sessions on KMC, strong referral links were developed between district hospitals and community health centres and community follow-up care of low birth weight (LBW) babies was strengthened. Statistics and success stories on KMC were used for advocacy at different levels (Bergh *et al.* 2012d).

### Country-level recommendations

On a national level, commitments from Ministries of Health or other supervisory bodies in support of KMC could assist in its promotion, and professional associations may be able to support training and professional development around KMC (Bergh *et al.* 2012c, 2014). Furthermore, countries with paid governmental leave for extended NICU stays or additional benefits for general maternity or paternity leave have aided in the uptake of KMC, demonstrating how paid parental leave could increase the feasibility of KMC (Calais *et al.* 2010; Blomqvist *et al.* 2013). Last, a cost and time effective alternative for KMC adoption would be to consistently incorporate the KMC method into medical staff training curriculum (Bergh *et al.* 2012b).

### Policy maker recommendations

Although the World Health Organization (WHO) guidelines provided an overarching outline of KMC (World Health Organization 2015), national policy makers have tried to make the policies more appropriate for their countries, taking into account available resources and cultural traditions (Bergh *et al.* 2012d). Although policy makers create policies and guidelines that affect HCWs and health facilities, they have very little direct interaction with these actors. To have a larger direct impact on implementation efforts, policy makers should create educational campaigns and advocate for expansion of KMC across all facilities within countries. Furthermore, if modifications of KMC are needed based on available resources and cultural traditions, communication between policy makers should facilitate these modifications. If countries and facilities have found solutions, sharing of knowledge could help facilitate consistent implementation in other facilities and countries facing similar barriers (Save the Children—Saving Newborn Lives Program 2011; Bergh *et al.* 2013).

### Strengths and limitations

This is one of the first systematic reviews to analyse qualitative research and program evaluations regarding KMC adoption from the health systems perspective. A major strength of our study lies in the comprehensive collection of studies from KMC research studies and implementation programmes. However, the generalizability of the results may be somewhat limited as the majority of studies came from areas with neonatal mortality rates (NMRs) <15 per 1000 live births. Although KMC is beneficial for all LBW or preterm infants, the intervention would be especially be impactful in low- and middle-income countries with high NMRs. Furthermore, we recognize that KMC protocols are distinct depending on the infant population, including preterm and LBW infants. The recommendations provided in our study will need to take into account context-specific limitations. Our review study summarizes the evidence and provides recommendations to support KMC implementation within health systems on a global level.

## Conclusion

KMC is a complex intervention, with unique barriers and enablers encountered at the HCW and facility levels within a health system. Understanding the challenges to implement KMC at each level of the health system and the interactions between levels of the health system provides recommendations for critical changes within a health system. Further research is needed to test models that address the barriers and support facilitators in order to promote and implement context-specific health system changes for greater KMC adoption.

## Supplementary Data

Supplementary data are available at *HEAPOL* online.

## Supplementary Material

Supplementary Table S1

Supplementary Table S2
