# Supplementary material for: Barriers and enablers of kangaroo mother care implementation from a health systems perspective: a systematic review
Source: Health Policy Plan. 2017 Aug 24;32(10):1466–75. doi: 10.1093/heapol/czx098 (PMC5886293; doi:10.1093/heapol/czx098)
Supplement: Supplementary Table S1 [file supplementary_table1_czx098.pdf]

Supplementary Table 1: Studies included in the Barriers and Enablers of Kangaroo Mother Care Implementation from a Health Systems Perspective: A Systematic Review (n=86)

Author	Year	Title	Country	Rural or Urban	Study design	Sample Size	Newborn Characteristics	KMC components	Onset of SSC	Hours per day KMC Provision	Number of days of KMC provision	Health care workers barriers and facilitators	Facilities barriers and facilitators	Policy and guideline barriers and facilitators
Abul-Fadl	2012	Evaluation of mothers' knowledge, attitudes, and practice towards the ten steps to successful breastfeeding in Egypt	Egypt	Mixed	Pop based surveillance, facility evaluation	1052 Mothers	All ages	SSC	N/A	N/A	N/A	X	X	-
Aliganyira	2014	Helping small babies survive: an evaluation of facility-based Kangaroo Mother Care implementation progress in Uganda	Uganda	Mixed	Facility evaluation, Focus group/interview	11 facilities	N/A	SSC	N/A	N/A	N/A	X	X	-
Araújo	2010	Mother Kangaroo Method: an investigation about the domestic practice	Brazil	Urban	Focus group/ interview	30 Parents	Premature, ≥2000g	N/A	Once eligible: N/A definition	5-6	N/A	X	X	-
Bergh	2013	Progress with the implementation of kangaroo mother care in four regions in Ghana	Ghana	N/A	Facility evaluation	38 Facilities	N/A	SSC, EBF, FU	Immediately after birth	N/A	N/A	X	X	X
Bergh	2003	Development of a conceptual tool for the implementation of kangaroo mother care	South Africa	Urban	Facility evaluation	2 Facilities	N/A	N/A	N/A	N/A	N/A	X	X	-
Bergh	2008	Scaling up kangaroo mother care in South Africa: 'on-site' versus 'off-site' educational facilitation	South Africa	Mixed	RCT	36 Facilities	N/A	N/A	N/A	N/A	N/A	X	X	-
Bergh	2012	Translating research findings into practice--the implementation of kangaroo mother care in Ghana	Ghana	N/A	Pop based surveillance, facility evaluation	38 Facilities	N/A	N/A	N/A	N/A	N/A	X	-	X
Bergh	2009	Scaling up Kangaroo Mother Care in Ghana	Ghana	N/A	Facility evaluation	4 Regions (out of 10)	N/A	N/A	N/A	N/A	N/A	X	X	-
Bergh	2012	Evaluation of Kangaroo Mother Care Services in Malawi	Malawi	N/A	Facility evaluation	14 facilities	N/A	N/A	N/A	N/A	N/A	X	X	X
Bergh	2012	Evaluation of Kangaroo Mother Care Services in Mali	Mali	N/A	Facility evaluation	7 Facilities	N/A	SSC, EBF, DC, FU	N/A	N/A	N/A	X	X	X

Bergh	2007	Retrospective Evaluation of Kangaroo Mother Care Practices in Malawian Hospitals	Malawi, South Africa	N/A	Facility evaluation	6 Facilities	N/A	N/A	N/A	N/A	N/A	X	X	-
Bergh	2012	Evaluation of Kangaroo Mother Care Services in Rwanda	Rwanda	N/A	Facility evaluation	7 Facilities	N/A	N/A	N/A	N/A	N/A	X	X	-
Bergh	2012	Evaluation of Kangaroo Mother Care Services in Uganda	Uganda	N/A	Facility evaluation	11 Facilities	N/A	N/A	N/A	N/A	N/A	X	X	X
Bergh	2014	Implementing facility-based kangaroo mother care services: lessons from a multi-country study in Africa	Malawi, Mali, Rwanda, and Uganda	Urban	Facility evaluation, Focus group/interview	39 facilities	N/A	SSC	N/A	N/A	N/A	X	X	X
Blencowe	2009	Safety, effectiveness and barriers to follow-up using an 'early discharge' Kangaroo Care policy in a resource poor setting	Malawi	Urban	Prospective cohort	272 Newborns	<2000g	N/A	Once eligible: N/A definition	N/A	N/A	-	X	-
Blomqvist	2013	Provision of Kangaroo Mother Care: supportive factors and barriers perceived by parents	Sweden	N/A	Focus group/ interview	76 Mother, 74 Fathers	28-33 weeks, 740-2920g	SSC	N/A	N/A	N/A	X	X	-
Blomqvist	2011	Swedish mothers' experience of continuous Kangaroo Mother Care	Sweden	Urban	Focus group/ interview	23 Dyads	All ages	SSC, EBF	N/A	N/A	N/A	X	X	-
Boo	2007	Short duration of skin-to-skin contact: Effects on growth and breastfeeding	Malaysia	Urban	RCT	126 Dyads	<1501g	SSC	Once eligible: N/A definition	1	10	X	X	-
Brimdyr	2012	A Realistic Evaluation of Two Training Programs on Implementing Skin-to-Skin as a Standard of Care	Egypt	N/A	Focus group/ interview	40 Nurses and HCWs	N/A	SSC	Immediately after birth	1	1	X	X	-
Calais	2010	Skin-to-skin contact of full term infants: an explorative study of promoting and hindering factors in two Nordic childbirth settings	Sweden, Norway	Urban	Focus group/ interview	117 Mother, 107 Fathers	Full term	SSC, DC, FU	Immediately after birth	N/A	N/A	-	-	X
Castiblanco López	2011	Vision of mothers in care of premature babies at home	Colombia	Urban	Focus group/ interview	8 Mothers	<36 weeks, 2320g	N/A	N/A	N/A	N/A	-	X	-

Charpak	2006	Resistance to implementing Kangaroo Mother Care in developing countries, and proposed solutions	15 developing countries	Mixed	Focus group/ interview	17 KMC co-ordinators, 15 Facilities	N/A	SSC, DC, FU	Immediately after birth	N/A	N/A	X	X	-
Chia	2006	The attitudes and practices of neonatal nurses in the use of kangaroo care	Australia	Urban	Focus group/ interview	34 Nurses	N/A	SSC	N/A	N/A	N/A	X	X	-
Colameo	2006	Kangaroo Mother Care in public hospitals in the State of Sao Paulo, Brazil: an analysis of the implementation process	Brazil	Mixed	Cross sectional	28 Facilities	LBW; N/A cutoff	N/A	Once eligible: N/A definition	N/A	N/A	X	X	-
Cooper	2014	Close to me: enhancing kangaroo care practice for NICU staff and parents	USA	Mixed	Pre-post	48 nurses and 101 parents	N/A	SSC	N/A	N/A	N/A	X	-	-
Crenshaw	2012	Use of a video-ethnographic intervention (PRECESS Immersion Method) to improve skin-to-skin care and breastfeeding rates	USA	N/A	Descriptive	261 Dyads	Full term	SSC	≤2min after birth	N/A	1	X	X	-
Dalal	2014	A cross-sectional study on knowledge and attitude regarding kangaroo mother care practice among health care providers in Ahmedabad district	India	Mixed	Cross sectional	145 HCPs	N/A	N/A	N/A	N/A	N/A	X	-	-
Dalbye	2011	Mothers' experiences of skin-to-skin care of healthy full-term newborns - A phenomenology study	Sweden, Norway	Urban	Focus group/ interview	20 Mothers	Full term	SSC	Immediately after birth	N/A	N/A	X	-	-
Darmstadt	2006	Introduction of community-based skin-to-skin care in rural Uttar Pradesh, India	India	Rural	Intervention	2063 Mothers	All ages	SSC	N/A	N/A	N/A	-	X	-

De Vonderweid	2003	Neonatal developmental care in Italian Neonatal Intensive Care Units	Italy	Mixed	Pop based surveillance	109 Facilities	N/A	N/A	N/A	N/A	N/A	X	X	X
Duarte	2001	Kangaroo mother care: experience report	Brazil	Urban	Focus group/ interview	1 Mother	Premature; N/A cutoff	SSC	N/A	N/A	38	-	X	-
Eichel	2001	Kangaroo care: Expanding our practice to critically iII neonates	USA	Urban	Facility evaluation	1 Facility	N/A	N/A	N/A	N/A	N/A	X	X	X
Eleutério	2008	The imaginary of mothers about experiencing the mother-kangaroo method	Brazil	Urban	Focus group/ interview	9 Mothers	Premature; N/A cutoff	N/A	N/A	N/A	N/A	-	X	-
Engler	2002	Kangaroo care: national survey of practice, knowledge, barriers, and perceptions	USA	Mixed	Facility evaluation	537 Facilities	N/A	N/A	N/A	N/A	N/A	X	X	-
Ferrarello	2014	Barriers to skin-to-skin care during the postpartum stay	USA	Urban	Focus group/ interview	15 Mother, 14 Nurses	N/A	SSC	N/A	N/A	N/A	-	-	X
Flynn	2010	Neonatal nurses' knowledge and beliefs regarding kangaroo care with preterm infants in an Irish neonatal unit	Ireland	Urban	Focus group/ interview	62 HCWs	N/A	N/A	N/A	N/A	N/A	X	-	-
Freitas	2007	Kangaroo Mother Method: newborn weight outcome	Brazil	N/A	Prospective cohort, descriptive	22 Newborns	N/A	N/A	N/A	N/A	N/A	-	X	-
Furlan	2003	Perception of parents in experiencing the kangaroo mother method	Brazil	Urban	Focus group/ interview	10 Parents	Premature; N/A cutoff	SSC	Once eligible: N/A definition	10;mean	N/A	-	X	X
Gontijo	2012	Evaluation of the implementation of Kangaroo Care by health administrators, professionals, and mothers of newborn infants	Brazil	Mixed	Focus group/ interview	293 Facilities	N/A	N/A	N/A	N/A	N/A	-	X	-
Gonya	2013	Factors associated with maternal visitation and participation in skin-to-skin care in an all referral level IIIc NICU	USA	Urban	Focus group/ interview	32 Mothers	<27 weeks	SSC	N/A	N/A	N/A	-	X	X

Haxton	2012	Implementing skin-to-skin contact at birth using the Iowa model: applying evidence to practice	USA	Urban	Intervention, qualitative	30 Mothers	All ages	SSC, EBF	Within one hour after birth	3	1	X	X	X
Heinemann	2013	Factors affecting parents' presence with their extremely preterm infants in a neonatal intensive care room	Sweden	N/A	Focus group/ interview	7 Mother, 6 Fathers	<27 weeks	SSC	N/A	N/A	N/A	-	X	-
Hendricks-Munoz	2010	Factors that influence neonatal nursing perceptions of family-centered care and developmental care practices	USA	Urban	Focus group/ interview	59 Nurses	N/A	SSC	N/A	N/A	N/A	X	-	-
Hendricks-Munoz	2013	Maternal and Neonatal Nurse Perceived Value of Kangaroo Mother Care and Maternal Care Partnership in the Neonatal Intensive Care Unit	USA	Urban	Focus group/ interview	143 Mother, 42 HCWs	<34 weeks	N/A	N/A	N/A	N/A	X	-	-
Hennig	2006	Health professional's knowledge and practices about KMC	Brazil	Mixed	Cross sectional	148 Doctors and nurses, 11 Facilities	LBW; N/A cutoff	N/A	Clinical stable	N/A	N/A	X	X	-
Higman	2015	Assessing clinicians' knowledge and confidence to perform kangaroo care and positive touch in a tertiary neonatal unit in England using the Neonatal Unit Clinician Assessment Tool (NUCAT)	England	Urban	Focus group/ interview	6 nurses and 51 clinicians	N/A	N/A	N/A	N/A	N/A	X	-	-
Hill	2010	Keeping newborns warm: beliefs, practices and potential for behavior change in rural Ghana	Ghana	Mixed	Focus group/ interview	635 Mother, 14 Villages	All ages	SSC	N/A	N/A	N/A	X	-	-
Hunter	2014	Newborn care practices in rural Bangladesh: Implications for the adaptation of kangaroo mother care for community-based interventions	Bangladesh	Rural	Focus group/ interview	121 participants	N/A	N/A	N/A	N/A	N/A	X	-	-

Johnson	2007	Factors influencing implementation of kangaroo holding in a Special Care Nursery	USA	Peri-urban/ Slum	Focus group/ interview	17 Nurses	N/A	N/A	N/A	N/A	N/A	X	X	-
Kambarami	2002	Caregivers' perceptions and experiences of 'kangaroo care' in a developing country	Zimbabwe	Urban	Focus group/ interview	40-48 mothers (4 focus groups with 10-12 participants per group)	LBW: N/A cutoff	N/A	N/A	N/A	N/A	-	X	-
Kostandy	2008	Kangaroo Care (skin contact) reduces crying response to pain in preterm neonates: pilot results	USA	N/A	RCT crossover	10 Newborns	30-32 weeks	SSC	30 min before heel stick	0.83	1	X	-	-
Kymre	2013	Balancing preterm infants' developmental needs with parents' readiness for skin-to-skin care: A phenomenological study	Sweden, Norway, Denmark	N/A	Focus group/ interview	18 Nurses	N/A	SSC	N/A	N/A	N/A	X	-	-
Lee	2012	Clinician perspectives on barriers to and opportunities for skin-to-skin contact for premature infants in neonatal intensive care units	USA	Mixed	Focus group/ interview	69 HCPs, 11 Facilities	N/A	SSC	N/A	N/A	N/A	X	X	-
Lemmen	2013	Kangaroo care in a neonatal context: parents' experiences of information and communication of nurse-parents	Sweden	N/A	Focus group/ interview	12 Families	24-35 weeks	SSC	N/A	N/A	N/A	X	-	-
Lincetto	1998	Impact of season and discharge weight on complications and growth of Kangaroo Mother Care treated low birth weight infants in Mozambique	Mozambique	Urban	Prospective cohort	246 Newborns	<2000g	SSC, EBF, DC, FU	Stabilized health condition, presence of a sucking reflex, thermoregulation, mother's condition enabling her to care for the LBW infant, cessation of the infant's need for IV therapy, oxygen, photo-therapy or feeding by NG tube	>20	N/A	X	X	-

Mallet	2007	[Skin to skin contact in neonatal care: knowledge and expectations of health professionals in 2 neonatal intensive care units]	France	N/A	Focus group/ interview	121 Doctors and paramedical staff	N/A	N/A	N/A	N/A	N/A	X	X	-
Morelius	2015	Neonatal nurses' beliefs about almost continuous parent-infant skin-to-skin contact in neonatal intensive care	Sweden	Urban	Survey	129 nurses	All newborns	N/A	N/A	N/A	N/A	X	-	-
Morelius	2012	Time of initiation of skin-to-skin contact in extremely preterm infants in Sweden	Sweden	Mixed	Pop based surveillance	520 Newborns	<27 weeks	SSC	N/A	N/A	N/A	X	-	-
Nahidi	2014	Opinions of the midwives about enabling factors of skin-to- skin contact immediately after birth: A descriptive study	Iran	Urban	Questionnaire	292 midwives	N/A	N/A	N/A	N/A	N/A	X	-	-
Namazzi	2015	Strengthening health facilities for maternal and newborn care: experiences from rural eastern Uganda	Uganda	Rural	RCT	20 health facilities	All newborns	SSC	N/A	N/A	N/A	X	X	-
Neu	1999	Parents' perception of skin-to-skin care with their preterm infants requiring assisted ventilation	N/A	Urban	Focus group/ interview	8 Mothers, 1 Father	Premature; N/A cutoff	SSC	N/A	1	2	X	X	-
Niela-Vilen	2013	Early physical contact between a mother and her NICU-infant in two university hospitals in Finland	Finland	Urban	Prospective cohort, qualitative	170 Mothers, 381 Staff	all NICU newborns	N/A	Immediately after birth	N/A	N/A	X	-	-
Nimbalkar	2014	Usage of EMBRACE(TM) in Gujarat, India: Survey of Paediatricians	India	Urban	Questionnaire	52 paediatrician s	N/A	N/A	N/A	N/A	N/A	X	-	-
Nyqvist	2008	Application of the baby friendly hospital initiative to neonatal care: suggestions by Swedish mothers of very preterm infants	Sweden	N/A	Focus group/ interview	13 Mothers	<32 weeks	SSC, DC, FU	N/A	N/A	N/A	X	X	X

Parmar	2009	Experience with Kangaroo mother care in a neonatal intensive care unit (NICU) in Chandigarh, India	India	Urban	Retrospective cohort	135 Newborns	26-37 weeks, 550-2500g	SSC	N/A	N/A	N/A	X	X	-
Pattinson	2005	Implementation of kangaroo mother care: a randomized trial of two outreach strategies	South Africa	Mixed	RCT	34 Facilities	N/A	N/A	N/A	N/A	N/A	-	X	-
Quasem	2003	Adaptation of kangaroo mother care for community-based application	Bangladesh	Urban	Focus group/ interview	35 Mothers	All ages	SSC	N/A	N/A	N/A	-	X	-
Ramanathan	2001	Kangaroo Mother Care in very low birth weight infants	India	N/A	RCT	28 Newborns	<1500g	N/A	Once eligible: N/A definition	≥4	N/A	X	-	-
Roller	2005	Getting to know you: mothers' experiences of kangaroo care	USA	N/A	Focus group/ interview	10 Mothers	32-37 weeks	SSC	N/A	N/A	N/A	X	X	X
Sacks	2013	Neonatal care in the home in northern rural Honduras: a qualitative study of the role of traditional birth attendants	Honduras	Rural	Focus group/ interview	48-72 TBAs (6 focus groups with 8-12 participants per group)	N/A	N/A	N/A	N/A	N/A	X	-	-
Santos	2013	Maternal perception of the skin to skin contact with premature infants through the kangaroo position	Brazil	Urban	Focus group/ interview	12 Mothers	Premature, LBW; N/A cutoff	SSC	N/A	N/A	N/A	-	X	-
Save the Children	2011	Scaling Up Kangaroo Mother Care, Report of Country Survey Findings	Ethiopia, Malawi, Mali, Mozambique, Nigeria, Tanzania, Uganda, Bolivia, Indonesia, Nepal, Vietnam	N/A	Facility evaluation	12 Countries	N/A	N/A	N/A	N/A	N/A	X	X	X

Silva	2014	Conhecimento de técnicos de enfermagem sobre o método canguru na unidade neonatal; Nursing technicians? knowledge of the Kangaroo-Mother Care method in the neonatal unit; Conocimiento de técnicos de enfermería sobre el método Canguro en la unidad neonatal	Brazil	Urban	Focus group/ interview	20 nursing technicians	N/A	N/A	N/A	N/A	N/A	X	-	-
Silva	2015	Nurses' adherence to the Kangaroo Care Method: support for nursing care management	Brazil	Urban	Focus group/ interview	8 nurses	N/A	N/A	N/A	N/A	N/A	X	-	-
Singh	2012	Utilization of postnatal care for newborns and its association with neonatal mortality in India: an analytical appraisal	India	Mixed	Case control	145662 Newborns, 810204 Mothers	All ages	N/A	N/A	N/A	N/A	-	-	X
Sinha	2014	Newborn care practices and home-based postnatal newborn care programme - Mewat, Haryana, India, 2013	India	Rural	Focus group/ interview	320 mothers, 61 Accredited Social Health Activists, 19 home visits	N/A	SSC, EBF	N/A	N/A	N/A	X	-	-
Sloan	2008	Community-based kangaroo mother care to prevent neonatal and infant mortality: a randomized, controlled cluster trial	Bangladesh	Rural	Cluster RCT	39888 Mothers	All ages	SSC	N/A	N/A	2; data available for first 2 days of life	X	-	-
Solomons	2012	Knowledge and attitudes of nursing staff and mothers towards kangaroo mother care in the eastern sub-district of Cape Town	South Africa	Urban	Cross sectional	30 Mothers, 15 Nurses	<2500g	N/A	N/A	N/A	N/A	X	X	-
Stikes	2013	Applying the plan-do-study-act model to increase the use of kangaroo care	USA	Urban	Focus group/ interview	56 Nurses	N/A	SSC	N/A	N/A	N/A	X	X	X

Strand	2014	Kangaroo mother care in the neonatal intensive care unit: staff attitudes and beliefs and opportunities for parents	Sweden	N/A	Facility evaluation	126 Staff	N/A	N/A	N/A	N/A	N/A	X	X	-
Toma	2007	Maternal perception of low birth weight babies before and following the implementation of the Kangaroo Mother Care in a public hospital, in the city of São Paulo, Brazil	Brazil	Urban	Focus group/ interview	41 Mothers	<2000g	N/A	Mean 18 days of life	N/A	N/A	-	X	-
Wahlberg	1992	A retrospective, comparative study using the kangaroo method as a complement to the standard incubator care	Sweden	Urban	Retrospective cohort	66 Dyads	Premature; N/A cutoff	SSC	N/A	N/A	N/A	X	X	-
Waiswa	2010	'I never thought that this baby would survive; I thought that it would die any time': perceptions and care for preterm babies in eastern Uganda	Uganda	Rural	Focus group/ interview	30 HCW and mothers, 16 Facilities	Premature; N/A cutoff	N/A	N/A	N/A	N/A	X	X	-
Wobi	2010	Report on the Kangaroo Mother Care Project at Komfo Anokye Teaching Hospital in Kumasi, Ghana	Ghana	Urban	Facility evaluation	2 Facilities	N/A	N/A	N/A	N/A	N/A	-	X	-
Zhang	2014	Evidence utilization project: implementation of kangaroo care at neonatal ICU	Singapore	Urban	Facility evaluation	1 ICU	Less than 34 weeks; Less than 1500 g	SSC	Once eligible: stable preterm or LBW babies, excluding infants with poor respiratory status, invasive lines, or parents who are depressed, not willing to do KMC, suffering from infectious skin disease on chest, unfit physically, or with flu-like symptoms.	At least 1 hour several times per day	N/A	X	X	-

Zwedberg	2015	Midwives' experiences with mother-infant skin-to-skin contact after a caesarean section: 'fighting an uphill battle'	Sweden	Urban	Focus group/ interview	8 midwives	N/A	N/A	N/A	N/A	N/A	X	-	-

Legend

X	Included in study
-	Not included in study
EBF	Exclusive Breast Feeding
DC	Discharge
FU	Follow Up
HCP	Health Care Provider
HCW	Health Care Worker
ICU	Intensive Care Unit
LBW	Low Birth Weight
N/A	Not available
NICU	Neonatal Intensive Care Unit
SSC	Skin to Skin Care
TBA	Traditional Birthing Attendant
