# Supplementary material for: Barriers and enablers of kangaroo mother care implementation from a health systems perspective: a systematic review
Source: Health Policy Plan. 2017 Aug 24;32(10):1466–75. doi: 10.1093/heapol/czx098 (PMC5886293; doi:10.1093/heapol/czx098)
Supplement: Supplementary Table S2 [file supplementary_table2_czx098.pdf]

Supplementary Table 2. Facility, Regional, and Country-level Recommendations

Level	Country	Arising Themes	Recommendations
**Facility**	Malawi	• **Improve Intermittent KMC protocols**<br>• **Involve ANC units**<br>• **Better record keeping**	Intermittent KMC should be more systematic. Build in more checklists for KMC treatment in an effort to ensure continuity. Improve record keeping at all levels. Improved communications with ANC units to better prepare mothers for LBW infants. Regular staff rotations.
	Mali	• **Improve Intermittent KMC protocols**	Encourage more systematic practice of intermittent KMC. Create opportunity to encourage mothers to practice KMC more diligently. Strengthen existing KMC services.
	Uganda	• **Improve discharge policies**<br>• **Formalized Social Support**	Specify discharge criteria and who is in charge of those policies. Officially support companions in order for a mother to better perform KMC.
	Brazil	• **Formalized Social Support**	Family participation is essential for success, it should occur at facility and at home.
	USA	• **Continuous in-service training**	Support and troubleshoot problems as they appear for KMC. Continued in-service KMC training of staff and parents.
**Regional**	Multi-country (Africa)	• Behavior Change Communication materials	Tailored Behavior Change Communication materials for specific in-country situations such as brochures, pamphlets, posters and videos where feasible.
	Malawi	• **District Implementation Plans include a KMC budget**<br>• **Continuous in-service training**	District Implementation Plans should include a KMC budget and protocols for training CHWs (including in-service trainings). KMC community mobilization.
	Rwanda	• **KMC budgeted at all levels**<br>• **In-service training**<br>• **Strengthen follow-up**<br>• **Involve ANC units**<br>• **Community sensitization**<br>• Provision of meals for mothers	KMC is in the budget at all implementation levels. Training and regular supervision should be maintained. Coordination between community-HCs-district hospitals. Strengthen follow-up. Better defined roles and responsibilities of HCWs. KMC included as part of ANC education. Map out how KMC is included in different curricula. Community sensitization. Behavior change workshops. Advocate for health authorities to pay for mothers' meals.
	Mali	• **Continuous in-service training**<br>• **Communication between regional, district and community facilities**<br>• **Strengthen follow-up**<br>• **Involve ANC**<br>• **Community KMC**	Two-tiered KMC structure in the community and the facility including continuous in-service trainings. Pediatricians to visit district hospitals from regional facilities and communication between region and community HCs. Abolish user-fees for L&D services to increase use of skilled birth attendants. Better follow-up after discharge. Include KMC in ANC services. KMC success stories to advocate and promote. Extension of KMC to community (only 50% of babies born in facilities). "Piggy-back" on other activities to introduce KMC.
**Country**	Sweden/Norway	• Paid maternal and paternal leave	The right to paid parental leave in connection with the birth of a child; paid paternal leave of 2 weeks and paid maternal leave for 1 year.
	Mali	• Use of new accredited KMC training center by district, regional and country level implementers.<br>• KMC included as performance indicator for MoH activities	Clarify roles and responsibilities of players at different levels. Establish an implementation network at all levels (regional hospitals, national and regional directorates of health.) Use of new accredited KMC training center. Renewed and upfront commitment by the MoH for the implementation of KMC at all relevant HFs. Should include KMC as performance indicator for MoH activities. Engage systematically with professional associations and other bodies to include KMC in professional development programs.
	Malawi	• Strengthen pre-service KMC training<br>• Less turnover of medical personnel at national level	Less rotation of medical personnel at national level. Strengthen pre-service education in KMC.
	India	• **Community KMC**	National Rural Health Mission (NRHM) should include KMC. Village level accredited social health programs should promote active SSC in the community among pregnant women.

KMC: kangaroo mother care; ANC: antenatal care; LBW: low birth weight; CHW: community health worker; HC: health center; L&D: labor and delivery; MoH: Ministry of Health; HF: health facility; SSC: skin-to-skin contact
